# Evaluation of single domain antibodies as nuclear tracers for imaging of the immune checkpoint receptor human lymphocyte activation gene-3 in cancer

**DOI:** 10.1186/s13550-021-00857-9

**Published:** 2021-11-02

**Authors:** Q. Lecocq, P. Debie, J. Puttemans, R. M. Awad, L. De Beck, T. Ertveldt, Y. De Vlaeminck, C. Goyvaerts, G. Raes, M. Keyaerts, K. Breckpot, N. Devoogdt

**Affiliations:** 1 Laboratory for Molecular and Cellular Therapy, Department of Biomedical Sciences, Vrije Universiteit Brussel, Laarbeeklaan 103/E, 1090 Brussels, Belgium; 2 In Vivo Cellular and Molecular Imaging Laboratory, Department of Medical Imaging, Vrije Universiteit Brussel, Laarbeeklaan 103/K, 1090 Brussels, Belgium; 3 Myeloid Cell Immunology Laboratory, VIB Center for Inflammation Research, Brussels, Belgium; 4 Cellular and Molecular Immunology Laboratory, Vrije Universiteit Brussel, Brussels, Belgium; 5 Nuclear Medicine Department, UZ Brussel, Brussels, Belgium

**Keywords:** Immunotherapy, Immune checkpoint, Lymphocyte activation gene-3, Single-domain antibody, Nanobody, Molecular imaging

## Abstract

Recent advancements in the field of immune-oncology have led to a significant increase in life expectancy of patients with diverse forms of cancer, such as hematologic malignancies, melanoma and lung cancer. Unfortunately, these encouraging results are not observed in the majority of patients, who remain unresponsive and/or encounter adverse events. Currently, researchers are collecting more insight into the cellular and molecular mechanisms that underlie these variable responses. As an example, the human lymphocyte activation gene-3 (huLAG-3), an inhibitory immune checkpoint receptor, is increasingly studied as a therapeutic target in immune-oncology. Noninvasive molecular imaging of the immune checkpoint programmed death protein-1 (PD-1) or its ligand PD-L1 has shown its value as a strategy to guide and monitor PD-1/PD-L1-targeted immune checkpoint therapy. Yet, radiotracers that allow dynamic, whole body imaging of huLAG-3 expression are not yet described. We here developed single-domain antibodies (sdAbs) that bind huLAG-3 and showed that these sdAbs can image huLAG-3 in tumors, therefore representing promising tools for further development into clinically applicable radiotracers.

**Supplementary Information:**

The online version contains supplementary material available at 10.1186/s13550-021-00857-9.

## Introduction

Cancer immunotherapy has become a standard treatment in oncology owing to the success of immune checkpoint blocking drugs [1–3]. Particularly monocloncal antibodies (mAbs) that block CTLA-4, PD-1 or its ligand PD-L1 have proven benefit in various cancer types [4–8]. These mAbs work by reinvigorating exhausted anticancer T cells and other immune cells by releasing the brake on their activation status, proliferation and cancer-infiltrating capacity [9]. Yet, the response of cancer patients after treatment with immune checkpoint blocking drugs remains mixed and unpredictable. In fact, it was estimated in 2018 that only 12.5% of US patients across all cancer types showed an objective clinical responses to this type of treatment [10]. In addition to the low response rate, treatment with immune checkpoint blocking mAbs frequently results in immune related adverse events (irAEs), which can potentially affect every organ [11–13]. Therefore, it remains a key challenge to evaluate ongoing treatment responses and stratify patients that may or may not benefit from a particular treatment [14–17].

To address this challenge, tracers have been developed that allow non-invasive imaging of the tumor microenvironment (TME) using radiolabeled probes, such as Abs and Ab-derived proteins, against specific immunological markers [14]. Molecular imaging with such tracers has the potential of monitoring biological processes in the TME before, during and after treatment with immunotherapeutic agents. Ultimately, these methods could be used to identify patterns of response and even predict treatment outcome. As an example, use of PD-L1 expression as a biomarker for patient stratification has been suggested in several PD-1/PD-L1 therapy studies [18]. Molecular imaging with mAbs or sdAbs have been used to detect PD-L1 expression in patients’ tumors [15, 19–21]. A comparative study showed the potential of molecular imaging to better predict patient outcome after PD-1/PD-L1-blockade compared to conventional immunohistochemistry (IHC) [22, 23].

The immune checkpoint receptor LAG-3 has emerged as a target for the treatment of cancer patients [24–27]. The potential benefit of targeting LAG-3 in cancer therapy is exemplified in the RELATIVITY-047 study. Herein, Relatlimab, a mAb that blocks LAG-3 signaling, is being tested in combination with Nivolumab, an anti-PD-1 mAb, in a phase II/III clinical trial for treatment of metastatic melanoma patients [28–30]. It was reported at the annual 2021 conference of the American Society of Clinical Oncology that primary endpoints, i.e., progression-free survival (PFS), were reached in this trial. At a median follow-up of 13.2 months, PFS was 10.12 months for Relatlimab plus Nivolumab combination therapy compared to 4.63 months for Nivolumab monotherapy with a hazard ratio of 0.75. Early reports stated that irAEs doubled in patients treated with Relatlimab plus Nivolumab combination therapy (19%) compared to Nivolumab monotherapy (9%) yet were still far below the number of irAEs observed upon Ipilimumab (anti-CTLA-4 mAb) plus Nivolumab combination therapy (59%) in the CHECKMATE-067 trials [31]. At the ASCO 2021 conference, it was further reported that while 33.4% of patients experienced grade 3/4 events in the Nivolumab monotherapy arm, this increased to 40.3% in the Relatlimab plus Nivolumab combination therapy arm with concomitant increase of 5.4% treatment discontinuation in the latter arm [28, 30]. Notably, it was shown that patients scoring less than 1% LAG-3 positivity in their tumor biopsies upon IHC evaluation, had a PFS (4.83 months) similar to patients receiving the monotherapy.

The preliminary results of the RELATIVITY-047 trial argue for the development of a tracer that allows repeated, whole body, non-invasive imaging of LAG-3 in cancer patients. This can guide medical doctors in their decision on if and when to start or, for that matter, discontinue the addition of LAG-3 blocking drugs (such as Relatlimab) to the currently approved Nivolumab therapy. So far, tracing of LAG-3 expressing human lymphocytes within human tumors in immunodeficient mice has been achieved using a zirconium-89 (^89^Zr) radiolabeled fully human anti-huLAG-3 Ab (REGN3767) and positron emission tomography (PET) [32]. However, as described for PET imaging of PD-L1 with ^89^Zr-labeled Atezolizumab [22], ^89^Zr-labeled anti-huLAG-3 antibody (REGN3767) only obtained optimal tumor-to-blood ratios days to 1 week after injection. This delay between tracer administration and imaging is undesired and can be circumvented when using tracers with a considerable smaller size. Currently, the ^89^Zr-labeled REGN3767 is evaluated for LAG-3 imaging in patients with advanced solid cancers that are treated with Cemiplimab, an anti-PD-1 antibody that, like REGN3767, was developed by Regeneron (NCT04706715).

SdAbs represent antibody fragments derived from heavy chain only Abs (HCAbs) found in members of the *Camelidae* family that have shown merit for PET-mediated imaging of cancer markers (e.g., HER2) [33] and immune cell receptors (e.g., MMR) [34] in the clinic. Previously we reported on the development of a sdAb-based PET-tracer targeting PD-L1 on cancer and immune cells [15, 21, 35, 36]. This experience encouraged the generation of sdAbs targeting LAG-3. We reported that mouse LAG-3 (moLAG-3)-specific sdAbs can be used to quantitatively and noninvasively image moLAG-3 expression on tumor-infiltrating lymphocytes at baseline and and after induction by PD-1-blocking therapy, showing predictive value for subsequent LAG-3 blocking therapy [16, 37]. In this study, we screened our LAG-3 specific sdAb library for sdAbs that bind huLAG-3 at high specificity and affinity. These sdAbs were labeled with 99 m-Technetium (^99m^Tc) and their biodistribution in healthy mice and mice bearing huLAG-3 expressing tumors was studied using single photon emission computed tomography (SPECT/CT) imaging. This effort led to the selection of a lead compound that is ready for clinical translation to a PET-tracer.

## Methods

### Mice, cell lines and reagents

Female, 6 to 12-week-old C57BL/6 or NU(NCr)Foxn1nu mice were purchased from Charles River (Ecully, France). Approval by the Ethical Committee for Laboratory Animals of the Vrije Universiteit Brussel was granted prior to execution of the experiments (ethical dossiers 15-214-1). All animal studies were performed in accordance with the European guidelines for animal experimentation. The HEK293T cells were obtained from the American Type Culture Collection (Molsheim Cedex, France). This cell line was cultured in Dulbecco’s modified Eagle’s medium (Sigma-Aldrich, Zwijndrecht, Belgium) supplemented with 10% fetal bovine serum (Harlan, Horst, The Netherlands), 2 mmol/L L-Glutamine (Sigma-Aldrich), 100 U/mL penicillin and 100 µg/mL streptomycin (Sigma-Aldrich). 2D3 cells were cultured in Iscove’s modified Dulbecco’s medium (Thermo Fisher Scientific, Aalst, Belgium) supplemented with 10% FBS, 2 mM L-Glutamine, 100U/mL penicillin and 100 µg/mL streptomycin. The TC-1 mouse lung epithelial cell line was provided by T.C. Wu (Johns Hopkins University, Baltimore, Maryland, USA) and cultured in Roswell Park Memorial Institute 1640 medium (Sigma-Aldrich), supplemented with 10% fetal clone I serum (Harlan), 2 mM L-Glutamine, 100 U/mL penicillin, 100 µg/mL streptomycin, 1 mmol/L sodium pyruvate and non-essential amino acids (Sigma-Aldrich), 12.5 mM D(+)-glucose, 1 mM Geneticin (G418), 5 mM HEPES and 50 μM β-mercaptoethanol. Recombinant huLAG-3 protein (Fc Chimera) used for Biacore surface plasmon resonance (SPR) studies was obtained from R&D Systems (2319-L3) (Bio-Techne, Abingdon, UK). Bovine serum albumin (BSA) was purchased from Sigma-Aldrich.

### Generation, production, characterization of anti-huLAG-3 sdAbs

Two llamas were subcutaneously immunized by the VIB Nanobody Service Facility (Brussels, Belgium) 6 times with a weekly interval with a mix containing 100 μg recombinant human LAG-3-Fc and mouse LAG-3-Fc proteins (R&D Systems, cat. 2319-L3 and 3328-L3). Gerbu LQ#3000 was used as adjuvant. Forty days later, blood was collected, and lymphocytes enriched for total RNA extraction. cDNA was synthesized from VHH encoding sequences using PCR. The amplicons were used as a source to create two sdAb-phage display libraries in the pMECS phagemids as described previously [38]. These libraries were phage-displayed and put through four rounds of biopanning on huLAG-3 or moLAG-3 recombinant protein that was immobilized on immunosorbent plastic. Next, freeze–thaw periplasmic extracts of individual clones were made and tested in ELISA on huLAG-3, moLAG-3 and control-Fc (human IgG1 Fc or mouse IgG2A Fc) fusion proteins. The different huLAG-3-specific sdAb clones were identified by sequencing. The immune library screening, production, characterization and radiolabeling of anti-huLAG-3 sdAbs were performed as described for moLAG-3 [16].

### Generation of huLAG-3 expressing cell lines

Mouse lung epithelial TC-1 cells were lentivirally transduced to express huLAG-3 (TC-1-huLAG-3) using an approach similar to that described for moLAG-3 [16]. In short, gBlocks were designed to contain the 1575 bp genetic code for huLAG-3 (CDS of NM_002286.6) and purchased from Integrated DNA Technologies. The sequences were flanked by 20 nucleotides that allow creating overhangs with the donor lentiviral transfer vector pHR’, which together with the packaging plasmid pCMVΔR8.9 and the VSV.G encoding plasmid pMD.G (gifts from D. Trono, University of Geneva, Switzerland) were used for production of lentiviral vectors. The production of lentiviral vectors by HEK293T cells and the transduction of 2D3 and TC-1 cells was performed as described [39]. 2D3 and TC-1 cells transduced with lentiviral vectors harboring huLAG-3 are referred to as 2D3-huLAG-3 and TC-1-huLAG-3 cells respectively.

### Flow cytometry

The process of staining cell surface markers with specific fluorescently labelled-antibodies was previously described [16]. 2D3 and TC-1 cells and their huLAG-3^+^ variants were incubated with 500 nM of selected anti-LAG-3 sdAbs in PBS plus 0.5% BSA for 60 min at 4 °C. R3B23, a sdAb that binds the 5T2 multiple myeloma idiotype was used as a control [40]. Mouse anti-HIS_6_-tag antibodies (Biorad, Belgium, clone AD1.1.10) were used as primary antibodies for staining of HIS_6_-tagged sdAbs. A phycoerythrin (PE) labeled anti-mouse IgG antibody (BD biosciences, clone A85-1) was used as secondary antibody to visualize sdAb binding using flow cytometry. A Peridinin-chlorophyll-protein (PerCP)-eFluor710 or PE-labeled antibody specific for huLAG-3 (eBiosciences, clone 3DS223H) was used in flow cytometry to evaluate huLAG-3 expression on 2D3 and TC-1 cells. Stained cells were acquired on a LSR Fortessa flow cytometer (BD Biosciences). Flow cytometry data were analyzed, and mean fluorescence intensity (MFI) was calculated using the FlowJo X® software (Tree star, Inc., Ashland, OR, USA).

### Radiolabeling of sdAbs

^99m^Tc-tricarbonyl [^99m^Tc(H_2_O)_3_(CO)_3_]^+^ was complexed with the C-terminal HIS_6_-tag of the sdAbs as described previously [16]. The former was performed using the Isolink® labeling kit (Mallinckrodt Medical BV). The ^99m^Tc-labeled sdAbs were purified by gel filtration on a PBS pre-equilibrated NAP-5 column (GE Healthcare) to remove uncomplexed (^99m^Tc(H_2_0)_3_(CO)_3_)^+^ and by filtration through a 0.22 μm filter (Millipore) to remove aggregates. The radiochemical purity was determined by instant thin layer chromatography (iTLC) before and after purification, with 100% acetone as the mobile phase.

### Biodistribution analysis of anti-huLAG-3 sdAbs in healthy and tumor-bearing mice

Imaging was performed as described [15]. Briefly, NU(NCr)Foxn1nu female mice (6–12 weeks, Charles River) were injected in the right or left hind leg with 10E6 wild type TC-1 or TC-1-huLAG-3 cells, respectively. Tumor-bearing mice or female C57BL/6 healthy mice (6–12 weeks, Charles River) were injected intravenously under isoflurane anesthesia (2.5% with an oxygen flow of 1L/min), with 5 µg of ^99m^Tc-labeled anti-huLAG-3 sdAbs with an average coupled radioactivity of 58.1 ± 12.2 MBq. SPECT/CT imaging was performed 1 h later on mice anesthetized with ketamine hydrochloride (75 mg/kg) and medetomidine (1 mg/kg) (Richter Pharma AG) using the Vector+/CT MiLABS scanner (MILabs). Medical Image Data Examiner and HOROS medical imaging viewer were used for image analysis. A region of interest (ROI) was assigned over the liver and activity within that ROI was calculated using quantification software. Eighty minutes after injection of ^99m^Tc-labeled anti-huLAG-3 sdAbs in tumor-bearing mice, mice were euthanized, tissues were isolated and counted using the Wizard2 gamma-counter (PerkinElmer, USA). Radiotracer uptake in tissue was corrected for decay and calculated as percentage of injected activity per gram tissue (%IA/g).

### Statistics

Statistical analyses were performed with GraphPad Prism software (version 7.2). Data are represented as mean ± standard deviation (SD). *p*-values were calculated using the Kruskal–Wallis test with Dunn's post hoc correction. Statistical results are indicated as *p < *0.05*; **p < *0.01*; ***p < *0.001*; ****p < *0.0001*; n.s. not significant.

## Results

### Selection of 16 sdAbs out of 114 that specifically bind the huLAG-3 protein

Llamas were immunized with recombinant moLAG-3 and huLAG-3 proteins to raise sdAbs that specifically bind huLAG-3. RNA extracted from the lymphocytes of these llamas was used to amplify the sdAb repertoire by PCR. This sdAb repertoire was cloned in a phagemid vector to generate an immune sdAb library in *E. coli*. Phage-displayed sdAbs were evaluated over four rounds of biopanning for their ability to bind recombinant moLAG-3 or huLAG-3 proteins immobilized on immunosorbent plates. A total of 114 sdAbs were selected after screening of the immune phage library as sdAbs that bound either moLAG-3 or huLAG-3. Cross-reactive sdAbs, meaning sdAbs that show binding to both moLAG-3 and huLAG-3, were not detected. Periplasmic extracts from positive scoring clones on huLAG-3 were analyzed in ELISA for binding to recombinant huLAG-3 protein, resulting in 37 sdAbs that were confirmed to specifically bind huLAG-3 (data not shown).

**Fig. 1:**
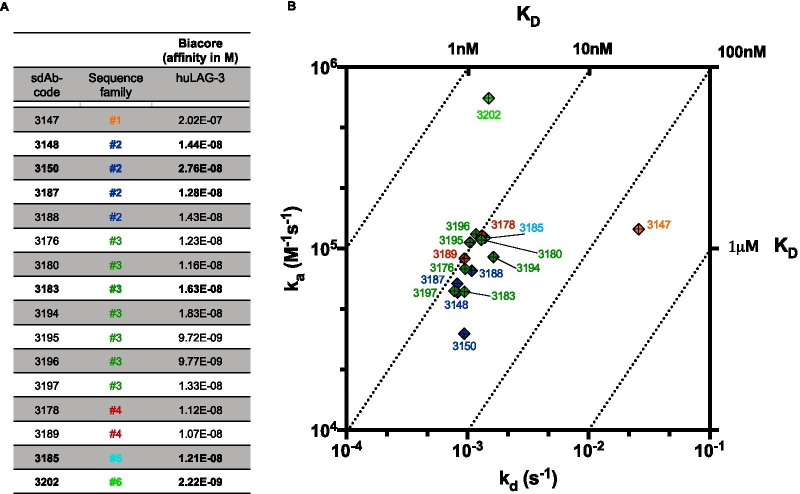
SPR analysis of anti-huLAG-3 sdAbs. **A** The table shows the code, sequence family and affinity of the sixteen selected sdAbs as determined by SPR on immobilized huLAG-3-Fc. **B** RaPID plot illustrating the association (*k*_a_) and dissociation (*k*_d_) values for each of the sdAbs, determined via affinity analysis using the biacore T100 instrument. Each sdAb, color-coded for family-homology, is indicated as a dot on the two-dimensional graph so that pairings with identical affinity (*K*_D_) values are located along iso-affinity diagonals

The sdAb-containing periplasmic extracts were further used to evaluate binding of the sdAbs to huLAG-3 on a chip (SPR, off-rate screening) or on cells (flow cytometry), showing that 16 sdAbs were able to bind huLAG-3 in both assays (data not shown), and these were subsequently produced and purified for further experimental evaluations. The amino acid sequences of the complementarity-determining regions (CDRs) CDR1, 2 and 3 were analyzed for these 16 sdAbs, showing that these belonged to 6 distinct sequence families (Fig. 1a). Analysis of the SPR results allowed comparison of the kinetic rate and affinity constants for the interaction between the sdAbs and huLAG-3, showing that the sdAbs’ affinity ranged from 1.07E−8 to 2.22E−9 Molar (M) with the exception of sdAb 3147 (2.02E−7M) (Fig. 1a,b).

**Fig. 2:**
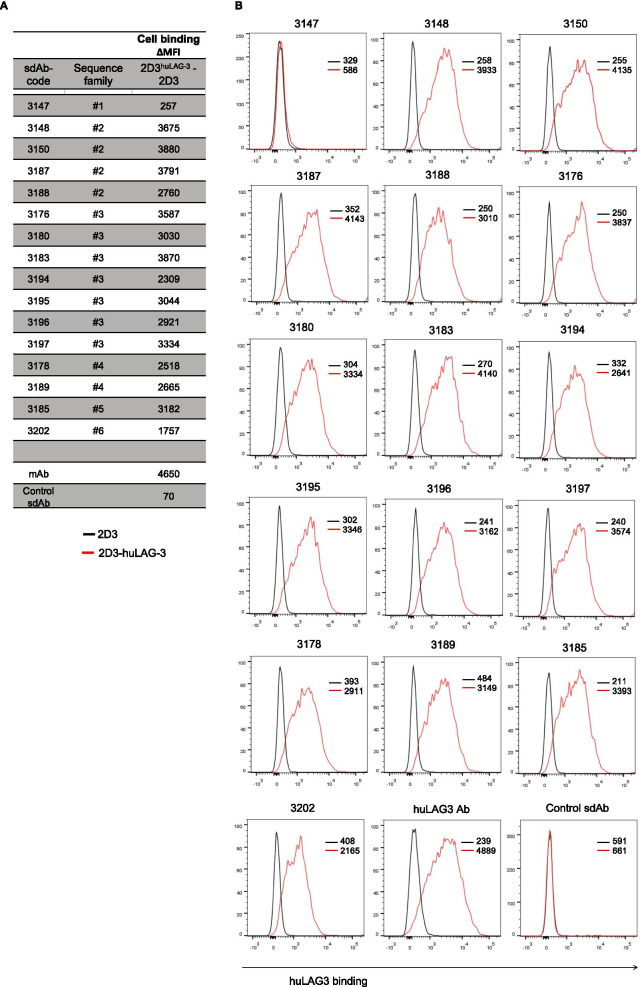
Binding characteristics of selected anti-LAG-3 sdAbs on huLAG-3 expressing 2D3 cells. **A** The table shows the code, sequence family and the binding of the sdAb to 2D3 cells versus 2D3-huLAG-3 cells, calculated using the MFI determined in flow cytometry. **B** Histogram showing binding of anti-huLAG-3 sdAbs on 2D3 (black line) or 2D3-huLAG-3 (red line) cells, measured using flow cytometry (*n* = 2)

To determine binding of the sdAbs to huLAG-3 on cells, we labelled 2D3 cells and their huLAG-3^+^ variants with the sdAbs and detected their binding in flow cytometry. The MFI obtained with 2D3 cells compared to 2D3-huLAG-3 cells was used to evaluate the sdAbs capacity to bind huLAG-3. sdAbs with high affinity for huLAG-3 as determined by surface plasmon resonance (SPR), showed strong binding to cell-bound huLAG-3 (Fig. 2a,b). Based on these results, the sdAbs highlighted in bold in Fig. 1a, i.e., sdAb 3148, 3150, 3183, 3185, 3187 and 3202 were selected for further analysis.

### Radiolabeling and biodistribution of ^99m^Tc-labeled sdAbs in healthy mice

**Fig. 3:**
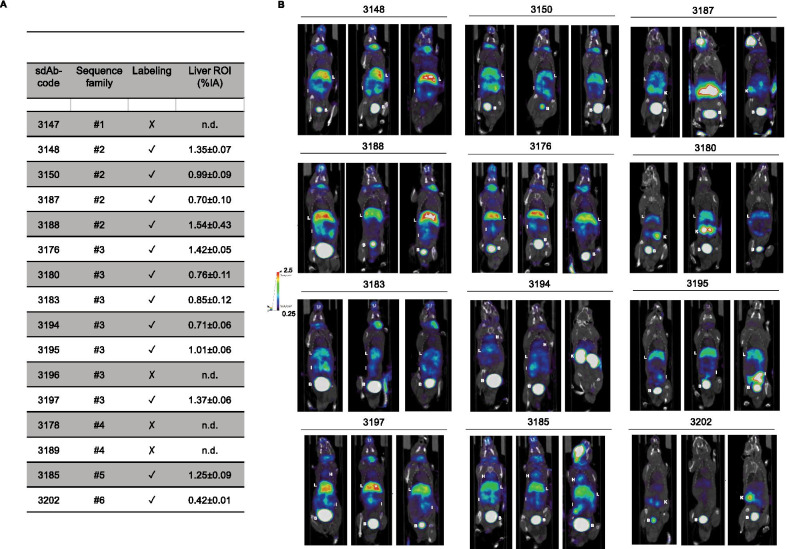
Biodistribution of selected anti-huLAG-3 sdAbs in healthy mice. **A** The table shows the code, sequence family, potential of labeling with ^99m^Tc, and uptake of the tracer in the liver. The successful or unsuccessful labeling of sdAbs with ^99m^Tc is depicted with ✓ or ✗ respectively. **B** SPECT/CT images generated 1 h after injection of ^99m^Tc-labeled sdAbs in healthy C57BL/6 mice (*n* = 3). K = kidney, L = liver, H = heart, I = intestines, B = bladder

To initiate imaging experiments, the efficiency of complexation of [^99m^Tc]Tc-tricarbonyl on the HIS_6_-tag of purified sdAbs was tested. Labeling with ^99m^Tc failed for sdAb 3147, the only sdAb of family 1; sdAb 3178 and 3189, two sdAbs of family 4 and sdAb 3196, one of the 6 sdAbs of family 3, while the radiochemical purity of the remaining ^99m^Tc-labeled sdAbs was over 98% as assessed by iTLC (Fig. 3a). To monitor non-specific uptake of ^99m^Tc-labeled sdAbs, we evaluated the biodistribution of these sdAbs in healthy C57BL/6 mice using SPECT/CT imaging, which was performed 1 h after intravenous injection of the ^99m^Tc-labeled sdAbs (Fig. 3b). We analyzed SPECT/CT images using quantification software and calculated the activity in the liver, as a measure of non-specific uptake. Hepatic signals were generally low, but still with marked differences between the various sdAbs (Fig. 3a). Of all sdAbs, ^99m^Tc-labeled sdAb 3202 demonstrated the lowest background.

### Specific huLAG-3 targeting of 99mTc-labeled sdAbs in tumor-bearing mice

**Fig. 4:**
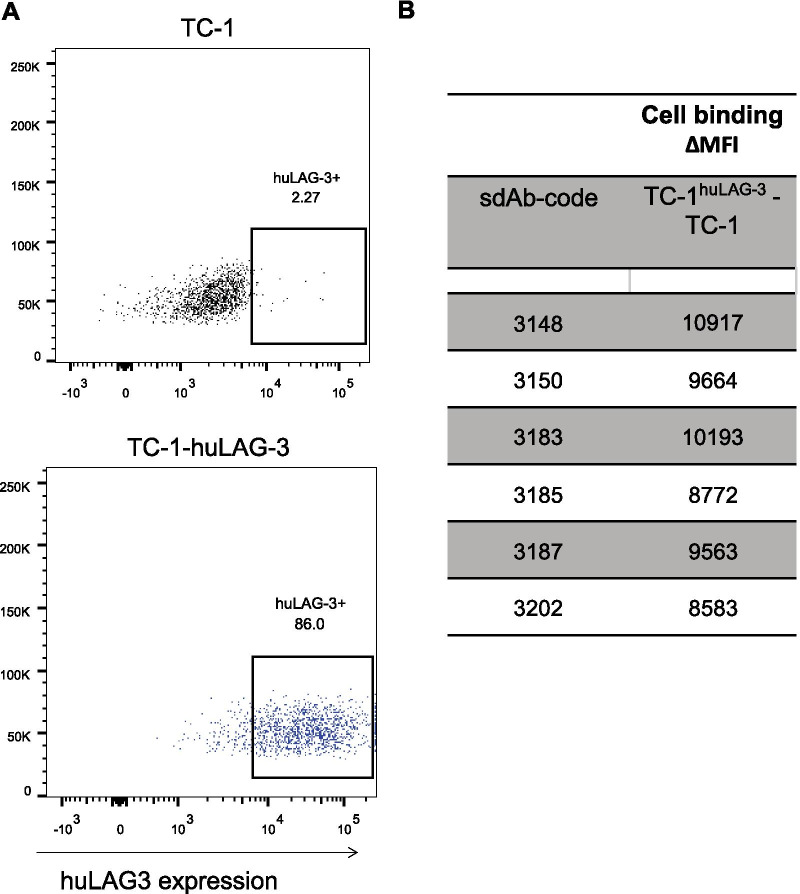
Expression of huLAG-3 and its binding by selected sdAbs on lentivirally engineered TC-1 cells. **A** Representative flow cytometry results, showing staining of TC-1 cells (black line) or TC-1-huLAG-3 cells (blue line) with an antibody specific for huLAG-3. **B** The MFI of TC-1 cells was used to determine the fold increase in MFI when these sdAbs bound to TC-1-huLAG-3 cells (*n* = 2)

**Fig. 5:**
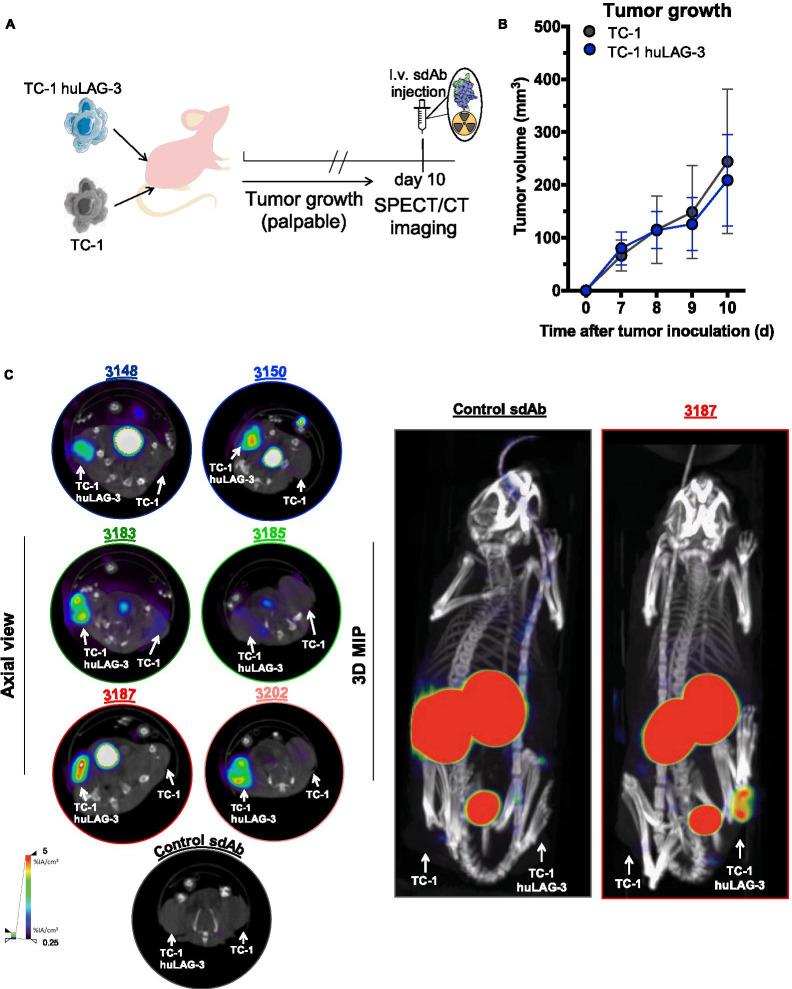
Visualization of huLAG-3 in mice bearing TC-1 and TC-1-huLAG-3 tumors using SPECT/CT imaging. **A** Schematic representation of the study design. **B** TC-1 and TC-1-huLAG-3 cells were injected subcutaneously at opposite hind legs of NU(NCr)Foxn1nu mice and allowed to grow tumors up till day 10. The graph shows the tumor volume in function of time as mean ± SD (*n* = 3). **c** Images of the SPECT/CT scans performed to evaluate the utility of selected ^99m^Tc-labeled sdAbs to detect huLAG-3 expressed in tumors. The arrows indicate the tumors on the images (*n* = 3). *MIP* = *Maximum Intensity Projection*

**Fig. 6:**
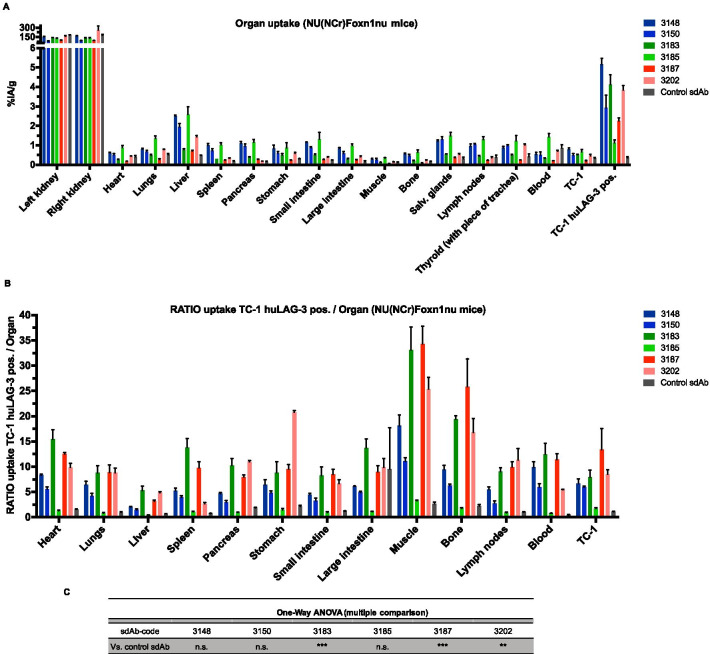
SdAb-mediated SPECT/CT imaging in TC-1-huLAG-3 tumor bearing mice shows that sdAb 3187 generates high signal-to-background ratios enabling detection of huLAG-3 in the tumor. **A** Results of ex vivo *γ*-counting analysis of uptake in isolated organs (expressed in %IA/g and as mean ± SD (*n* = 3)). **B** The graph shows the ratio in uptake in the TC-1-huLAG-3 positive tumor compared to the indicated organs. **C** The table shows the statistical results of comparing TC-1-huLAG-3 positive tumor/organ ratio values between control sdAb and huLAG-3 targeting sdAbs, confirming significant differences for sdAbs 3187 and 3202

The following parameters were considered when selecting sdAbs 3148, 3150 and 3187 (family 2), sdAb 3183 (family 3), sdAb 3185 (family 5) and sdAb 3202 (family 6) for further analysis in tumor bearing mice: (i) sdAb sequence, (ii) affinity (SPR), (iii) strength of binding to cell-bound huLAG-3 and (iv) low non-specific liver uptake. To evaluate whether the selected ^99m^Tc-labeled sdAbs can target huLAG-3 in tumors, TC-1-huLAG-3 lung epithelial cells (Fig. 4a) were transplanted in the hind leg of NU(NCr)Foxn1nu mice after in vitro confirmation of binding of the selected sdAbs to huLAG-3 expressed on these cells (Fig. 4b, Additional file 1: Fig. S1). Non-modified TC-1 cells were transplanted on the opposite hind leg of these mice (Fig. 5a). Tumor growth was monitored for 10 days, showing similar growth kinetics for TC-1 and TC-1-huLAG-3 tumors (Fig. 5b). SPECT/CT imaging 1 h after tracer administration showed that the selected ^99m^Tc-labeled sdAbs accumulated in TC-1-huLAG-3 tumors yet not in TC-1 tumors with little accumulation in peripheral organs except for the kidneys and bladder (Fig. 5c). Ex vivo analysis of radiotracer uptake in tumors and peripheral organs (Fig. 6a) corroborated selective accumulation of the sdAbs in TC-1-huLAG-3 tumors, although to the least extent for sdAb 3185. sdAb 3185 displays slightly elevated retention throughout different organs and the blood, thereby pointing at non-specific uptake. The highest specific tumor uptake was observed for sdAbs 3148 and 3183. To better appreciate signal-to-noise ratios, the uptake in LAG-3 expressing tumors was divided by different background organs and tissues, as displayed in Fig. 6b, showing the highest values for sdAb 3187, with sdAb 3183 and 3202 on second and third place respectively. The ratios for these sdAbs were significantly different from control sdAb (Fig. 6b,c). Therefore, sdAb 3187 was chosen as the lead sdAb for further development into a PET-tracer that allows non-invasive imaging of the huLAG-3.

## Discussion

Therapy with mAbs that block CTLA-4, PD-1, or PD-L1, is increasingly becoming standard of care for various solid tumor types. Yet, objective evaluation of treatment responses in US cancer patients with diverse cancer types shows that only a minority of patients benefit from this therapy [10]. Moreover, it has been reported that only a fraction of these responsive patients will remain progression-free 5 years after initiating therapy [41]. Therefore, there is an unmet need for additional interventions that can promote response to or reverse resistance to therapy with immune checkpoint blocking mAbs that target CTLA-4, PD-1 or PD-L1. Since LAG-3 is often co-expressed with PD-1 on tumor-infiltrating lymphocytes [42, 43], it has been proposed that blockade of LAG-3 in conjunction with blockade of PD-1 could be promising to increase treatment response [26, 44]. Preclinical studies [37] and the preliminary results of the RELATIVITY-047 trial [28–30] confirm this hypothesis and suggest that stratification of patients based on LAG-3 expression allows predicting therapy outcome. As such there is a need to develop tracers that allow noninvasive dynamic whole body imaging of LAG-3.

To date, a ^89^Zr-labeled anti-LAG-3 antibody (REGN3767) has been developed and is under clinical evaluation to image LAG-3 expression in patients with diffuse large B cell lymphoma (NCT04566978) and patients with solid cancer that are treated with the PD-1 blocking mAb, Cemiplimab (NCT04706715). It is however expected that optimal tumor-to-blood ratios will only be obtained days to 1 week after injection, as was described for PET imaging of PD-L1 with ^89^Zr-labeled Atezolizumab [22]. This delay between tracer administration and imaging is undesired and can be circumvented when using tracers with a considerable smaller size. Despite being smaller, sdAbs are able to bind epitopes with similar affinity and specificity as compared to mAbs. Moreover, sdAbs have a high thermal and chemical stability, which allow the use of labeling methods and chemical modifications in conditions that are incompatible with mAbs [36]. Due to their fast blood clearance, sdAbs are able to generate high-contrast images as early as 1 h post-injection. Moreover, rapid clearance of sdAbs allow the use of fast-decaying radioisotopes, which will benefit the patients' and healthworkers' safety [45]. The ability of sdAbs to be used for same-day imaging is particularly interesting for the characterization of immune checkpoints since they are dynamically expressed on immune cells [46]. The information gathered will guide clinicians to quickly formulate optimal immune checkpoint blockade treatment regimens.

Therefore, we generated sdAbs that bind LAG-3, as sdAbs are small-sized antibody fragments that allow rapid generation of high contrast images of molecular markers expressed on cancer cells and cancer-infiltrating immune cells without toxicity or evidence of immune responses against the sdAb-based tracer [47], as shown in clinical trials using sdAbs for PET imaging of HER2 [33] and MMR [34]. SPECT/CT scans with the selected ^99m^Tc-labeled lead sdAb (3187) showed specific and high-contrast detection of huLAG-3 on the surface of tumor cells in vivo, 1 h after intravenous injection of the tracer. This confirms that same day imaging, similar to the clinical practice with fluorine-18 (^18^F)-labeled fluorodeoxy-glucose, is feasible, as also shown for other sdAb-based tracers, albeit labeled with gallium-68 (^68^Ga), another PET compatible radiolabel [21, 36, 48]. PET is preferred over SPECT imaging in clinical practice as it is more sensitive and provides higher resolution.

Yet, we opted for labeling of sdAbs with ^99m^Tc by complexing their C-terminal HIS_6_-tag with [^99m^Tc]Tc(CO)_3_ and SPECT/CT imaging of tumor bearing mice because this labeling strategy is straightforward and has little impact on the sdAb’s biodistribution [49]. In contrast, labeling of sdAbs with PET isotopes such as ^68^Ga or ^18^F requires different radiochemistry procedures that can be considered more challenging [21, 48, 50, 51]. Both ^68^Ga and ^18^F have a decay half-life that matches with the sdAbs’ pharmacokinetic properties. While ^68^Ga is a radiometal that needs a chelator like NOTA for conjugation to the sdAb under mild conditions, ^18^F is a radiohalogen that is covalently bound to another atom and therefore requires harsh conditions for sdAb labeling. As a result, ^68^Ga is at this point most studied in the context of sdAb-based PET imaging. Notably, NOTA is a bifunctional chelator that is usually conjugated on the primary amino groups of lysines in the sdAb’s structure. When the sdAb has a lysine in one of its CDRs, NOTA conjugation could impact on the affinity of the sdAb for its antigen. This is however dependent on individual sdAb-antigen interactions, as certain sdAbs with lysines in the CDRs are not impacted by chelator conjugation to lysines [21]. Nevertheless, as sdAb 3187 has a lysine in its CDR3 region, the impact of NOTA chelation and ^68^Ga-labeling on the binding capacity on this huLAG-3 specific sdAb should be further investigated. The ease of producing ^18^F has instigated research into milder labeling strategies, such as use of [^18^F]SFB that also couples with lysines, therefore might impact on sdAb properties as well. Nonetheless, biologically this strategy is interesting, as it has been observed that kidney retention can be reduced significantly [52]. To avoid the use of lysines, site-specific approaches are of interest. Site-specific labeling via the Sortase-A enzyme is possible, however, requires genetic modification of the sdAb, inserting the Sortase-A recognition site “LPETG” at the C-terminus [21, 53]. Similarly, installation of a cysteine at the sdAb C-terminus allows site-specific conjugation using maleimide chemistry [36, 54]. So-called “click” chemistry could be a viable alternative for radiofluorination of sdAbs yet requires further investigation to address the full potential of the various available methods to generate ^18^F-sdAb-based PET-tracers [55, 56].

In conclusion, we report that sdAb 3187 is a promising probe for imaging of huLAG-3 in solid tumors, ready for translation to a PET-tracer for clinical use. Extrapolating from ongoing clinical trials with a ^68^Ga-labeled anti-HER2 sdAb breast cancer PET-tracer [33] and an anti-MMR sdAb macrophage PET-tracer [48], we are hopeful that the future clinical anti-LAG-3 sdAb PET-tracer will be safe, sensitive and conveniently provide a whole-body picture of huLAG-3 expression levels in a same-day imaging procedure with acceptable dosimetry levels.

## Supplementary Information

**Additional file 1:** Evaluation of sdAb binding to huLAG-3 modified TC-1 cells. Histograms showing binding of the indicated sdAbs to TC-1 (black line) or TC-1-huLAG-3 (blue line) cells as detected in flow cytometry.

## Data Availability

Data will be made available upon request.

## Abbreviations

huLAG-3: Human lymphocyte activating gene-3
PD-1: Programmed death
PD-L1: Programmed death ligand-1
sdAbs: Single domain antibodies
mAbs: Monoclonal antibodies
TME: Tumor microenvironment
IHC: Immunohistochemistry
PFS: Progression-free survival
^89^Zr: Zirconium-89
HCAbs: Heavy chain only Abs
moLAG-3: Mouse lymphocyte activating gene-3
^99m^Tc: Technetium-99m
PET: Positron emission tomography
SPECT: Single photon emission computed tomography
SPR: Surface plasmon resonance
BSA: Bovine serum albumin
PE: Phycoerythrin
PerCP: Peridinin-chlorophyll-protein
MFI: Mean fluorescence intensity
iTLC: Instant thin-layer chromotography
ROI: Region of interest
%IA/g: Percentage injected activity per gram
SD: Standard deviation
M: Molar
^68^Ga: Galium-68
