# Supplementary material for: Evaluation of single domain antibodies as nuclear tracers for imaging of the immune checkpoint receptor human lymphocyte activation gene-3 in cancer
Source: EJNMMI Res. 2021 Nov 2;11:115. doi: 10.1186/s13550-021-00857-9 (PMC8563901; doi:10.1186/s13550-021-00857-9)
Supplement: Supplementary file 1 — Additional file 1: Evaluation of sdAb binding to huLAG-3 modified TC-1 cells. Histograms showing binding of the indicated sdAbs to TC-1 (black line) or TC-1-huLAG-3 (blue line) cells as detected in flow cytometry. [file 13550_2021_857_MOESM1_ESM.docx]

**
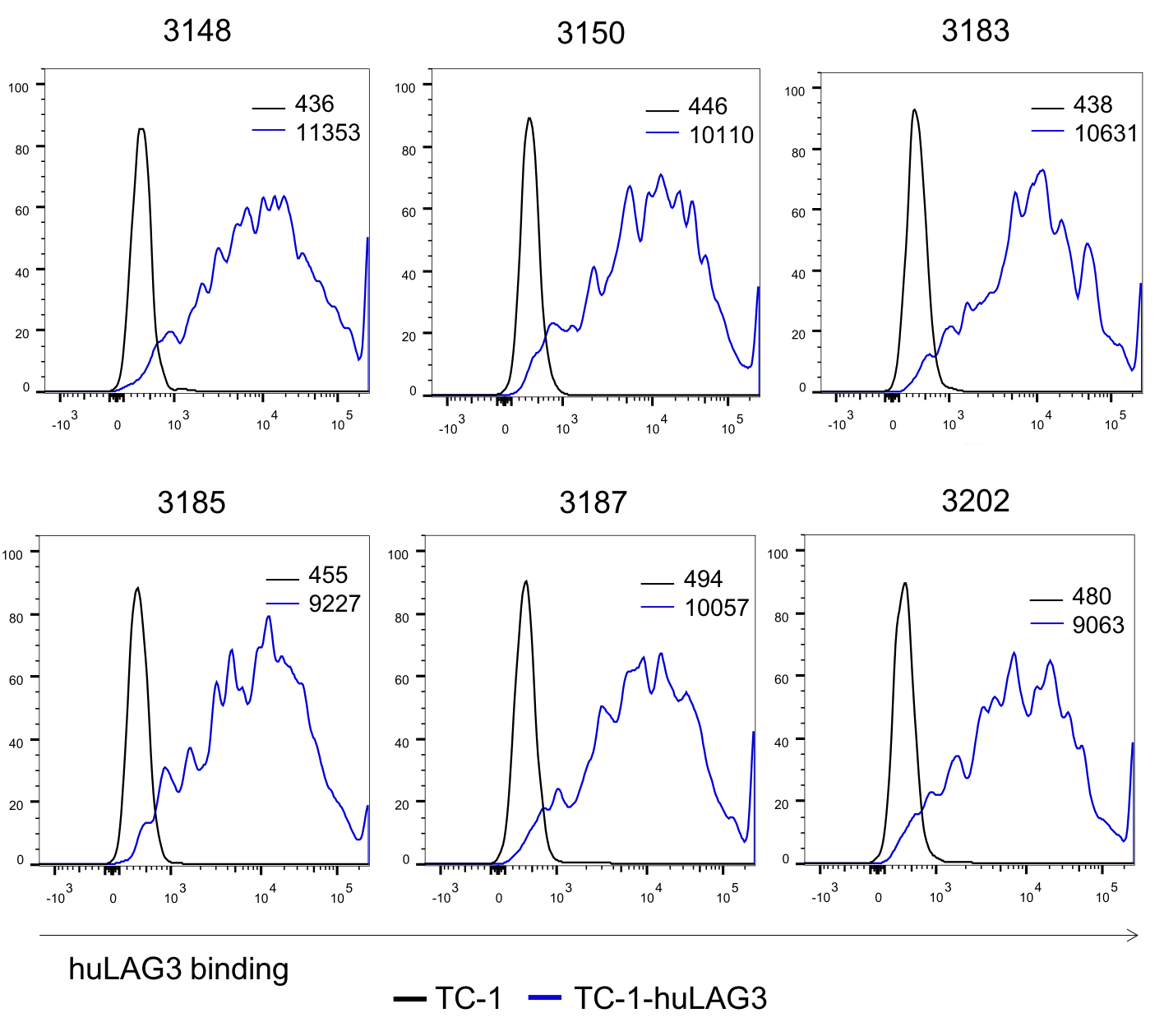
**

**Supplementary Figure and figure legend**

**Figure S1:** Evaluation of sdAb binding to huLAG-3 modified TC-1 cells. Histograms showing binding of the indicated sdAbs to TC-1 (black line) or TC-1-huLAG-3 (blue line) cells as detected in flow cytometry.
